# Insights into the Red Seaweed *Asparagopsis taxiformis* Using an Integrative Multi-Omics Analysis

**DOI:** 10.3390/plants14101523

**Published:** 2025-05-19

**Authors:** Min Zhao, Tomas Lang, Zubaida Patwary, Andrew L. Eamens, Tianfang Wang, Jessica Webb, Giuseppe C. Zuccarello, Ana Wegner-Thépot, Charlotte O’Grady, David Heyne, Lachlan McKinnie, Cecilia Pascelli, Nori Satoh, Eiichi Shoguchi, Alexandra H. Campbell, Nicholas A. Paul, Scott F. Cummins

**Affiliations:** 1 School of Science, Technology and Engineering, University of the Sunshine Coast, Maroochydore, QLD 4558, Australia; t_l059@student.usc.edu.au (T.L.); zpatwary@usc.edu.au (Z.P.); aeamens@usc.edu.au (A.L.E.); twang@usc.edu.au (T.W.); jessica.webb@research.usc.edu.au (J.W.); athepot@grooteaqua.com.au (A.W.-T.); c.ogrady@uq.edu.au (C.O.); dheyne@usc.edu.au (D.H.); lachlan.mckinnie@research.usc.edu.au (L.M.); cpascelli@aims.gov.au (C.P.); acampbe1@usc.edu.au (A.H.C.); npaul@usc.edu.au (N.A.P.); 2 Centre for Bioinnovation, University of the Sunshine Coast, Maroochydore, QLD 4558, Australia; 3 School of Health, University of the Sunshine Coast, Maroochydore, QLD 4558, Australia; 4 School of Biological Sciences, Victoria University of Wellington, Wellington 6012, New Zealand; joe.zuccarello@vuw.ac.nz; 5 Marine Genomics Unit, Okinawa Institute of Science and Technology Graduate University, Onna 904-0495, Okinawa, Japan; norisky@oist.jp (N.S.); eiichi@oist.jp (E.S.)

**Keywords:** *Asparagopsis taxiformis*, draft nuclear genome, multi-omics, resource

## Abstract

The red seaweed *Asparagopsis taxiformis* (Bonnemaisoniaceae, Rhodophyta) produces a bioactive natural product, bromoform, which, when fed to ruminant livestock, can eradicate methane emissions. However, to cultivate enough *A. taxiformis* to produce a yield that would have a meaningful impact on global greenhouse gas emissions, we need to advance our current understanding of the biology of this seaweed species. Here, we used both a domesticated diploid tetrasporophyte (>1.5 years in culture) and wild samples to establish a high-quality draft nuclear genome for *A. taxiformis* (lineage 6 based upon phylogenetic analyses using the *cox2-3* spacer). The constructed nuclear genome is 142 Mb in size (including 70.67% repeat regions) and was determined to encode for approximately 10,474 protein-coding genes, including those associated with secondary metabolism, photosynthesis, and defence. To obtain information regarding molecular differences between cultured and wild tetrasporophytes, we further explored differential gene expression relating to their different growth environments. Cultured tetrasporophytes, which contained a relatively higher level of bromoform compared to wild tetrasporophytes, demonstrated an enrichment of regulatory factors, such as protein kinases and transcription factors, whereas wild tetrasporophytes were enriched for the expression of defence and stress-related genes. Wild tetrasporophytes also expressed a relatively high level of novel secretory genes encoding proteins with von Willebrand factor A protein domains (named rhodophyte VWAs). Gene expression was further confirmed by proteomic investigation of cultured tetrasporophytes, resulting in the identification of over 400 proteins, including rhodophyte VWAs, and numerous enzymes and phycobiliproteins, which will facilitate future functional characterisation of this species. In summary, as the most comprehensive genomic resource for any *Asparagopsis* species, this resource for lineage 6 provides a novel avenue for seaweed researchers to interrogate genomic information, which will greatly assist in expediating production of *Asparagopsis* to meet demand by both aquaculture and agriculture, and to do so with economic and environmental sustainability.

## 1. Introduction

Our ability to identify and harness the potential of biodiversity to meet the environmental challenges posed by agriculture is critical for future food production and security and, while in parallel, mitigate the effects of climate change [1,2]. This notion is particularly pertinent for ruminant livestock, including cattle and sheep, that collectively “belch” the methane produced by their gut microbes, with the produced methane accounting for more than 6% of global greenhouse gas emissions [3]. Methane has a global warming potential 28 times that of carbon dioxide over a 100-year period, and in Australia, around 15% of total greenhouse gas emissions come from agriculture, of which two-thirds is accounted for by the methane produced by ruminants [4]. Therefore, reducing methane emissions from agriculture represents one of the most significant challenges for the sustainability of extant agricultural systems, not just in Australia, but globally.

Recently, the use of seaweed (marine macroalgae) as feed additives for cattle and sheep was investigated [5], with this research demonstrating that one species of tropical-to-warm-temperate red seaweed, *Asparagopsis taxiformis* (Delile) Trevisan, can largely eliminate enteric methanogenesis in cattle rumen fluid when added in dried form at 2% of the organic matter. This effect was almost exclusively related to one halogenated natural product known as bromoform or tribromomethane [5]. The research was based on the natural product knowledge of the genus *A. taxiformis* and its evaluation against other marine and freshwater macroalgae [6]. While many seaweeds and microalgae release *de novo* bromoform [7], *A. taxiformis* is unique in its capacity to store high concentrations of bromoform (and other halogenated compounds) in specialised gland cells, reaching up to 4% of dry weight. This contrasts with other red algae like *Laurencia* and *Delisea*, which store halogenated metabolites in similar distinct cellular structures (e.g., corps en cerise), yet lack comparable bromoform accumulation [8]. This natural source of bromoform is so concentrated that the glands are a prominent feature of *A. taxiformis* cells under light microscopy, with no other seaweed or plant species known to localise or accumulate bromoform in its tissues to this extent [8].

With regulations against the manufacture, import and export of some of the most effective synthetic additives for mitigating methane emissions [9], local researchers and industry have instead heavily invested in finding natural alternatives [10]. Many seaweeds other than the species of *Asparagopsis*, and more specifically, the natural products contained within them, have antimicrobial activity. Moreover, while some seaweed additives can alter the ruminant microbiome [11], and others can also reduce methane production; none are comparable to *A. taxiformis* with activity at <2% feed dry matter, leading to undetectable methane production [5]. *A. taxiformis* is the only seaweed that can be used as a high-efficacy feed additive for ruminants because it specifically targets the archaea portion of the gut microbiome without impacting fermentation [5].

International interest in the use of a natural feed additive in feedlots, including New Zealand, the United States, and countries in Europe, has shifted the question from “what additive?” to “how can we produce *A. taxiformis* at scale?” There needs to be significant investment in infrastructure and knowledge to meet the global demand for this seaweed feed additive and to retain the efficacy of the biomass under large-scale production. Controlled, large-scale aquaculture is the only means to deliver a product of consistent quality and quantity (i.e., a minimum content of bromoform that is free from impurities). However, we know that the content of the halogenated natural products in natural populations of seaweeds is variable, with many direct and indirect effects influencing the production of such products, including biosynthetic pathway variation [12], genetic and environmental components [13], through to ecological interactions with microbes [14,15]. For seaweeds such as *A. taxiformis* with a triphasic diplohaplontic heteromorphic life cycle (i.e., dioecious haploid gametophyte, diploid carposporophyte, and diploid tetrasporophyte phases), there are also life cycle differences in halogenated natural product production [16]. The biological and ecological variation in the production of natural products needs to be understood and addressed before companies can sensibly invest in commercialising the production of *A. taxiformis*.

By understanding the biosynthetic pathways of bromoform and other halogenated natural products in *A. taxiformis* and evaluating differential gene expression across the life cycle, critical information can be gained regarding the biology and ecology of the production of bromoform in *A. taxiformis* under different culture conditions and environments. To date, two draft genomes of *A. taxiformis* (unknown lineages) have been used to identify a bromoform-producing haloperoxidase gene cluster [termed the marine bromoform biosynthesis (*mbb*) locus], leading to their recombinant production for functional analysis [17]. Genetic information is critical for fast-tracking the development of this new aquaculture species and for setting the parameters for what is possible within this biological system and guiding investment in research and natural product development. The availability of a high-quality genome can provide the foundation resource to support such multi-level investigations [18], the benefits of which have been demonstrated in other red seaweeds, including *Chondrus crispus* Stackhouse, *Gracilariopsis chorda* (Holmes) ohmi, *Porphyra umbilicalis* Kützing, *Pyropia haitanensis* (T.J. Chang and B.F. Zheng) N.Kikuchi and M.Miyata [2,11,18,19]. For *A. taxiformis,* genomic sequencing has already been useful for the elucidation of a bromoform biosynthesis locus [11,17], although limited genomic features were reported in this instance. The addition of transcriptomic and proteomic information enables an in-depth multi-omic assessment of this red seaweed species. For example, the interaction between *A. taxiformis* and the coral *Astroides calycularis* (Pallas, 1766) was explored based on the microbiome and metabolomic data [20]. In other red seaweeds, a transcriptomic-derived protein database for *Eucheuma denticulatum* enabled the proteomic characterisation of extracellular proteins following soluble protein extraction [21]. In the red seaweed *Pyropia haitanensis*, a genome-derived protein database was used to document the proteins associated with molecular responses to various forms of environmental stress [22].

In this study, we present a draft assembly of the *A. taxiformis* (lineage 6) nuclear genome, sequenced from a cultured tetrasporophyte that was collected and domesticated from Queensland, Australia, where the first demonstration of the anti-methanogenic effects of the seaweed was conducted [6]. Here, we go on to report the genome features of *A. taxiformis* in comparison to the nuclear genomes of other red seaweed species, which may assist researchers in fundamental and applied investigations related to climate change mitigation, as well as agricultural and aquacultural production. The robustness of this resource was provided by the use of a multi-omic comparative investigative approach of both cultured and wild tetrasporophytes.

## 2. Results and Discussion

### 2.1. Phylogeny of A. taxiformis and Description of the Sample Used for Genomic Analysis

Our phylogenetic analysis of the *cox2-cox3* spacer, commonly used in intra-specific diversity studies in *Asparagopsis*, indicated that the sporophyte sample used in the genome sequencing (2019; submitted as NCBI GenBank accession number OP779373) is a member of lineage 6 (L6) of *A. taxiformis* [23] (Figure S1). This lineage is common in tropical Australia, including coastal regions of Queensland and Western Australia. Our sample is part of a strain designated the Great Barrier Reef strain [23], the strain used in feeding trials in Australia [24,25]. The genetic diversity of *Asparagopsis*, both *A. taxiformis* and *A. armata*, indicates that multiple distinct lineages are found in both species, which prove there may be many cryptic species within these morphological forms, although formal taxonomic changes have not been made [23,26]. While this tropical Australian lineage is the best studied in relation to its anti-methanogenic effects in livestock so far, the monophyly of all other taxonomically related *A. taxiformis* lineages, and including *A. armata*, suggests that the genomic information presented here will serve as an important resource and reference for future comparative studies on all other *Asparagopsis* genus species.

### 2.2. Genomic Features of Asparagopsis taxiformis (L6)

Here, we present the first draft genome for the red seaweed *A. taxiformis* (L6), which was determined to have a total size of 142 megabases (Mb). This high-quality nuclear genome for *A. taxiformis* provides the basis for subsequent multi-omics exploration of this lineage. The assembled genome of *A. taxiformis* (L6) contained 3308 contigs and a median contig length of 55,006 base pairs (bp) (Table 1). By comparison to the only other reported *A. taxiformis* assemblies [17], this assembly had a larger total assembly size (142 Mb vs. 75 Mb and 47 Mb), higher average contig length, and higher median contig length (N50). Based on the 255 eukaryotic BUSCO groups, we found 208 complete BUSCOs (81.6% complete genes), among which there were 183 complete single-copy BUSCOs. In addition, there were only 11 fragmented BUSCOs and 36 absent BUSCOs. Therefore, *A. taxiformis* (L6) had a better overall assembly compared to *A. taxiformis* Guam (GCA_018397955.1) and *A. taxiformis* California (GCA_018397975.1) (File S1a).

Approximately 70.87% of the *A. taxiformis* (L6) assembly was interspersed repeats, including 20.71% retroelements, 33.48% DNA transposons, and 0.21% rolling circle sequences, and the remaining 16.47% of identified repeats were grouped as unclassified repeats. The conserved gene structures collected from BUSCO were used for training in AUGUSTUS and predicted 10,863 putative genes in *A. taxiformis* (L6) genome (File S1b). These genes were further cleaned from microbial contaminants, which resulted in a final number of 10,474 *A. taxiformis* genes. In addition, we generated full-length genomes for the mitochondria (26,034 bp) and plastid (176,936 bp). By using sequence-based searching against our mitochondrial and plastid genomes, we predicted 50 mitochondrial genes and 227 plastid genes, respectively (File S1c). Among the 50 mitochondrial genes, we found 2 ribosomal RNA (rRNA) genes and 22 different transfer RNA (tRNA) genes.

**Figure 1:**
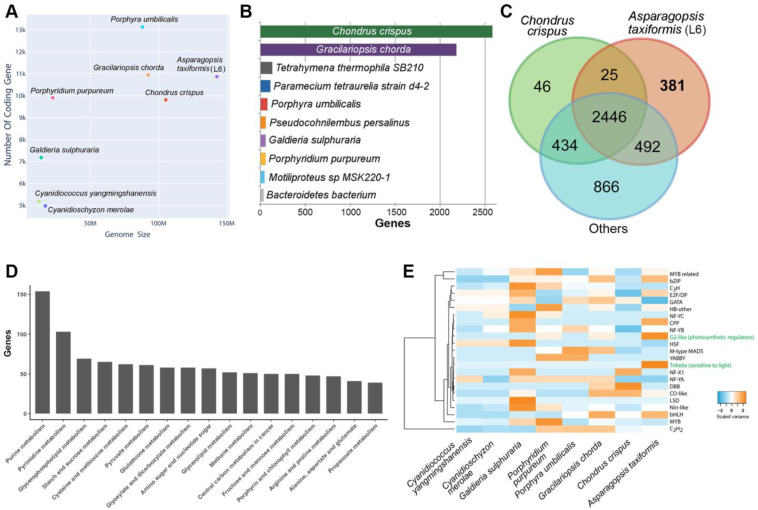
The *Asparagopsis taxiformis* (Lineage 6) draft genome and comparative functional overview. (**A**) Comparison of genome size and the number of protein-coding genes among eight red algal species with completed genome and proteome data. (**B**) The species distribution based on best-matched BLASTp (2.9.0) search results. (**C**) Shared Pfam entries among *A. taxiformis*, *C. crispus*, and the remaining six red algal genomes. (**D**) Number of genes associated with metabolism KEGG pathways (50 or more genes). (**E**) Interspecies comparison of transcription factors, where green font represents relatively large expansion in *A. taxiformis*.

We explored the comparative nuclear genome size and the number of protein-coding genes across eight red seaweeds with whole genome data available (Figure 1A). *Asparagopsis taxiformis* (L6) was revealed to have the largest genome at 142.472 Mb for the eight red seaweeds assessed, yet it ranked third in terms of the total number of protein-coding genes. For comparison, the *Porphyra umbilicalis* genome is predicted to encode 13,125 putative genes and to have a total genome size of 87.889 Mb [1]. In total, the *A. taxiformis* (L6) genome contained 6737 single exon (monoexonic) genes (64.32%), with monoexonic genes a commonly reported feature of seaweed protein-coding sequences. For example, in the red seaweed *C. crispus*, genes are remarkably compact, containing only 1.32 exons on average (i.e., many fewer than other organisms of similar genome size), and most genes (88%) are monoexonic [24].

### 2.3. Comparative and Functional Features of the A. taxiformis (L6) Genome

To provide a well-annotated genomics resource, we utilised a BLAST2GO pipeline, which is the most efficient approach to associate genes with proteins of known function. Of the 10,474 genes, there were 8409 genes (80.03%) with homologous matches to the NCBI non-redundant (NR) database based on an E-value of 0.00001. The similarity for each BLAST result was defined by division of the exact number of matches over the length of the matched sequence. The majority of matches had over 60% similarity (Figure S2A), and the species distribution showed that the highest identity of *A. taxiformis* genes was with *C. crispus* (2583 genes), followed by *G. chorda* (2182 genes) (Figure 1B), reflecting their evolutionary history at the order level [27]. Among the genes with NR homologues, 6950 genes (66.35%) were further mapped to gene ontology (GO) terms. By examining the top 10 GO terms, we found the most abundant categories consisted of cytosol and membrane genes (cellular components), peptide transport and protein phosphorylation encoding genes (biological processes), and protein and ATP binding encoding genes (molecular function) (Figure S3B).

Of the 10,474 *A. taxiformis* (L6) genes, 9074 genes contained a protein functional site and domain (assigned by InterPro annotations), while 5931 genes (56.63%) had Pfam protein family/domain mapping. By comparing the Pfam annotation among seven other red algae genomes (same species as Figure 1A), we found that *A. taxiformis* shared 2471 Pfam domains with *C. crispus* (supporting our BLAST analysis) and 2938 with the other red algae species (Figure 1C). Of interest, 381 *A. taxiformis* unique domains could be placed into categories of functional domains, protein families, and repeats based on InterPro annotations (Figure S2C). The most prominent of these, which included 186 *A. taxiformis* genes, was associated with the tryptophan-aspartic acid 40 (WD40) repeat functional domain: a domain superfamily that encompasses members of the beta-propeller domain family that are known to form interaction scaffolds in protein complexes [28]. Mapping *A. taxiformis* genes to the KEGG database showed that 5059 genes (48.3%) had KEGG orthologues, of which 1067 genes were categorised into “metabolism”. Of those with 40 or more genes, we found purine metabolism and pyrimidine metabolism contained over 80 *A. taxiformis* genes (Figure 1D), a finding that suggests that nucleotide hydrolysis, potentially as part of biomolecule production, is of high functional importance in *A. taxiformis*.

As a key regulator of gene expression, transcription factors (TFs) are central to all developmental processes of an organism. To explore the presence of TF families in the *A. taxiformis* genome, and for comparison to other red algae with sequenced genomes, we identified 145 TF families (File S1d) and performed a comparative genome-wide analysis of TFs in the seaweeds (Figure 1E). Similar to other seaweeds, TFs belonging to C2H2, bZIP, MYB-related, and bHLH families have the highest number of family members in *A. taxiformis*. Some TFs are located close to each other in the genome, where, for example, two MYB-related TF encoding loci were determined to be positioned immediately adjacent to each other. Such proximity of genes encoding members of the same gene family strongly infers that they are the product of a gene duplication event as part of the species-specific evolution of the *A. taxiformis* nuclear genome. In plants, MYB TFs are generally associated with plant development and the response of a plant to either biotic and abiotic stress [29]. The identification of such high numbers of MYB domain containing TFs infers that those members of this TF gene family potentially mediate similar responses to changes to the external environment in *A. taxiformis*. Interestingly, a single trihelix TF (TTF) was identified in *A. taxiformis* (L6), while no equivalent TTFs could be identified in the other seven red seaweed species assessed. A TTF protein has a highly conserved three-helix structure (helix-loop-helix-loop-helix) and has been shown to be responsive to light [30]; therefore, identification of a TTF only in *A. taxiformis* infers that this TF also occupies an important role in the ability of *A. taxiformis* to respond to changes to its external growth environmental.

**Figure 2:**
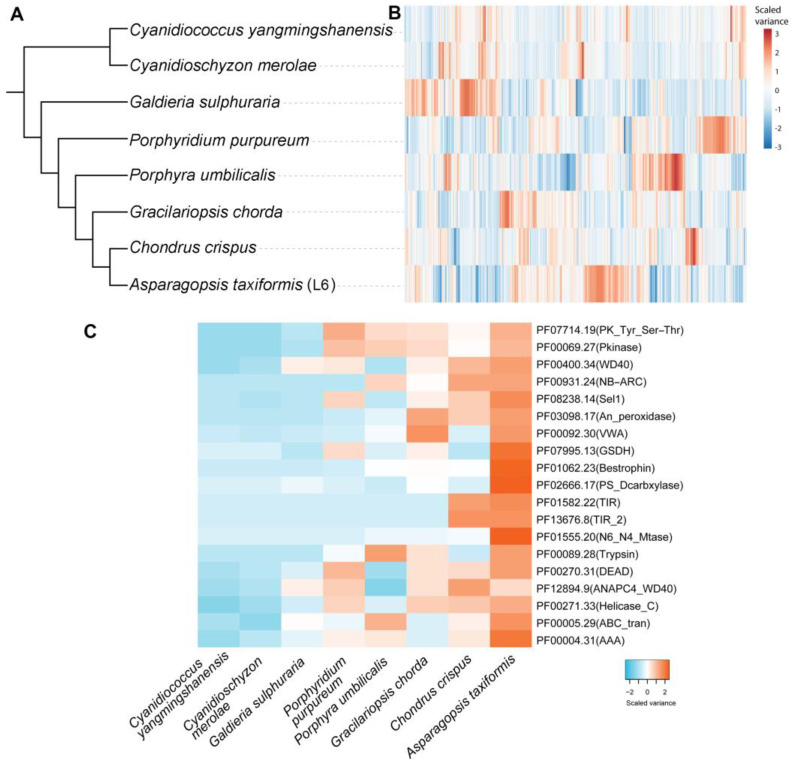
Comparative Pfam genomics of *Asparagopsis taxiformis* (L6) and seven other red seaweeds. (**A**) Phylogenetic tree constructed based on all the orthologous sequences inferred from OrthoFinder. (**B**) Heatmap showing the clustering of Pfam families based on the gene abundance in each species. Each row is a Pfam domain. (**C**) The top 20 Pfam domains that underwent a gene duplication event in *A. taxiformis* following branching from *Chondrus crispus*. The clustering in the heatmap was based on the number of genes for each Pfam entry (row) in the corresponding species (column).

Ortholog analysis was performed on eight red seaweed genome-derived proteome datasets (72,277 proteins) that had been subjected to all-against-all BLASTp analysis (E-value of 0.00001). This analysis clustered 56,813 proteins (78.6%) into 9841 orthologous groups, which were used to construct a species tree (Figure 2A,B). Among the orthologous groups, 2430 (11,578 proteins) were species-specific. Interestingly, 15,148 putative gene duplication events were identified among the 8 genomes, all of which are traced back to the node of the species tree on which these duplications occurred. For *A. taxiformis* (L6), there were 1359 non-terminal duplications. These were mapped to 1233 Pfam domains, and the number of genes in each protein family from each species was aligned with the species tree.

To better understand lineage-specific gene duplications, we mapped 4829 *A. taxiformis* genes suspected to be the product of duplication events to Pfam annotations to provide a comprehensive survey of gene duplication in *A. taxiformis* (e.g., polyploidy). Then we prioritised the Pfam domains by the number of duplicated genes (Figure 2C). Gene duplications often generate new gene copies, which may be under less selection pressure than the template gene. Therefore, these duplicated genes may acquire new expression domains and/or differing function, which forms one of the major driving forces in genome evolution [31]. Potentially the most striking feature was the 66 genes, which were determined to encode a protein product that harbours a N6_N4_Mtase domain in *A. taxiformis* (L6), compared to six, five, and four such genes in *C. crispus*, *P. umbilicalis* and *G. chorda*, respectively (Figure 2C). In terms of metabolic enzyme duplication, we identified 25 glucose/sorbosone dehydrogenase (GSDH) genes in the *A. taxiformis* (L6) genome, with this gene number again expanded in *A. taxiformis* from the number of GSDH genes identified in *P. purpureum* (1 gene), *P. umbilicalis* (5 genes), *G. chorda* (6 genes), and *C. crispus* (5 genes). In plants, GSDH is important for ascorbic acid metabolism to generate ascorbate precursors [32]. The phosphatidylserine decarboxylases (EC:4.1.1.65, PS_Dcarbxylase), which are known to catalyse the reaction: phosphatidyl-L-serine <=> phosphatidylethanolamine + CO_2_ [33], are prominent in *A. taxiformis* (L6), with 15 family members identified, compared to the other analysed red seaweeds that have a combined total of 9 phosphatidylserine decarboxylase genes. In summary, these duplicated genes which encode for enzymes known to function in biosynthesis pathways may provide clues to explore gene innovations in *A. taxiformis* (L6) required for the biosynthesis of ascorbate precursors and aminophospholipids.

### 2.4. A. taxiformis (L6) Cultured and Wild Sporophyte: An Integrative Omics Analysis

The availability of a high-quality draft genome provides the first comprehensive resource for the integrative omics-based analysis of *A. taxiformis* to determine how environmental conditions alter the bromoform content of sporophytes, as well as to link how changes to the expression of the genes, which encode the proteins of this biosynthesis pathway, differ between lab and wild cultivated *A. taxiformis*. Cultured *A. taxiformis* (L6) tetrasporophytes were maintained in largely sterile water at a constant temperature, light intensity, and salt level, whereas wild tetrasporophytes are exposed to constantly changing abiotic (e.g., conditions of temperature, light intensity, salt, and nutrient fluctuations) and biotic conditions (e.g., rabbitfish [34]); therefore, the genes associated with defence and stress resistance are likely to have significantly different levels of expression. In addition, tetrasporophytes cultured in the laboratory setting have not been exposed to the growth conditions that would promote their transition into reproductive tetrasporophytes, an artificial bottleneck to their developmental progression, which too would likely induce large gene expression changes in these two distinct populations of *A. taxiformis*.

**Figure 3:**
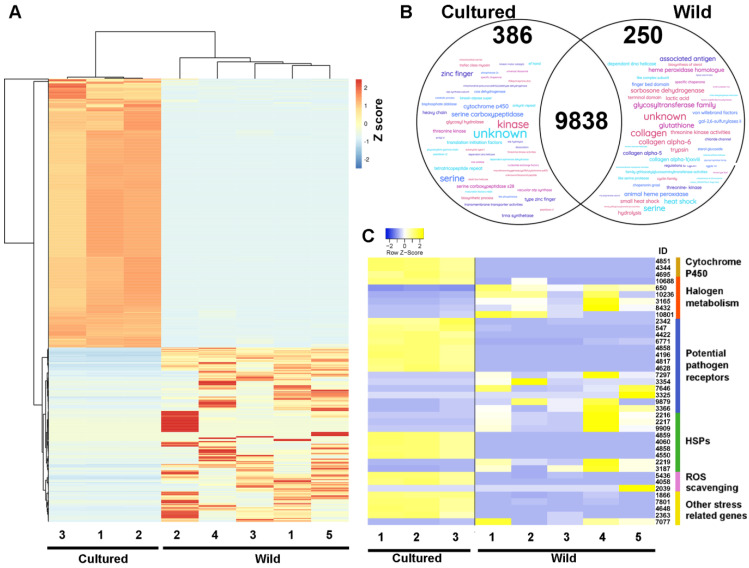
Summary of differential gene expression between *A. taxiformis* (L6) cultured and wild tetrasporophytes. (**A**) Heatmap with hierarchical clustering showing relative gene expression between cultured and wild tetrasporophytes. (**B**) Venn diagram shows those genes with greater than four-fold higher expression in cultured or wild tetrasporophytes and highlights (Word Cloud) their proposed function based on relative abundance of annotation. (**C**) Heatmap showing relative expression of genes significantly different between cultured and wild tetrasporophytes that are associated with stress and defence.

Differences in gene expression were therefore next investigated between cultured and wild tetrasporophytes of *A. taxiformis*. Overall, cultured tetrasporophyte total gene expression exhibited stronger clustering based on principal component analysis (PCA), a finding that readily highlighted the greater variation in gene expression in the wild samples (Figure S3). This may reflect the greater variability in growth conditions, such as temperature and light, that the wild sporophyte samples would have been exposed to compared to the controlled conditions of cultured tetrasporophytes. Of those genes expressed, 399 and 257 genes were identified as significantly differentially expressed (FDR *p* < 0.05, >±4 log_2_ fold-change) between cultured and wild tetrasporophytes, respectively (Figure 3A). In cultured tetrasporophytes, there was a relative enrichment of genes whose sequences aligned to enzymes (e.g., kinases, serine carboxypeptidases, and cytochrome P450s) and regulatory factors (e.g., zinc finger proteins and translation initiation factors) (Figure 3B). In addition, *cyclin* (*Ata*L6 *9753*), which encodes a protein well known for complexing with cyclin-dependent kinase (CDK-cyclin complexes) to regulate the progression of a cell through the cell cycle [25], was notably absent in cultured tetrasporophytes yet was highly expressed in wild tetrasporophytes (File S1e). This finding aligns well with our observed suppression of cultured sporophyte progression through to the reproductive stage of their development. We expect that more in-depth molecular comparative analysis of non-reproductive and reproductive (with sporangia) tetrasporophytes, including changes in CDK-cyclin complex coupling, could provide the necessary knowledge to manipulate sporophyte transitions in seaweeds, including *A. taxiformis* (L6), an area of study that should form a high-priority item of *A. taxiformis* research by our group in the future.

In wild tetrasporophytes, there was a relative enrichment of genes that were annotated as haloperoxidases, homologues of animal heme peroxidase-like proteins and heat shock proteins (HSPs), which fit within designated categories (based on *C. crispus* gene annotations [24]) related to defence and stress-resistance genes (Table 2 and Figure 3C). Therefore, the relative abundance of these transcripts in wild tetrasporophytes formed an interesting finding. The HSP family (including, but not limited to, HSP20, HSP70, and HSP100) are well known for their role in facilitating protein folding, assembly, translocation, and degradation during normal cellular homeostasis [35]. However, they additionally have the ability to direct the stabilisation of proteins and to assist their refolding under stress conditions [24]. We identified that several stress-responsive HSPs were upregulated in wild tetrasporophytes, specifically those members subcategorized within the HSP20 family (molecular weights ~20 kDa). This finding aligns well with research in the red seaweed *Pyropia yezoensis*, where small-sized HSPs were upregulated during high temperature, oxidative stress, or altered copper levels [36].

**Figure 4:**
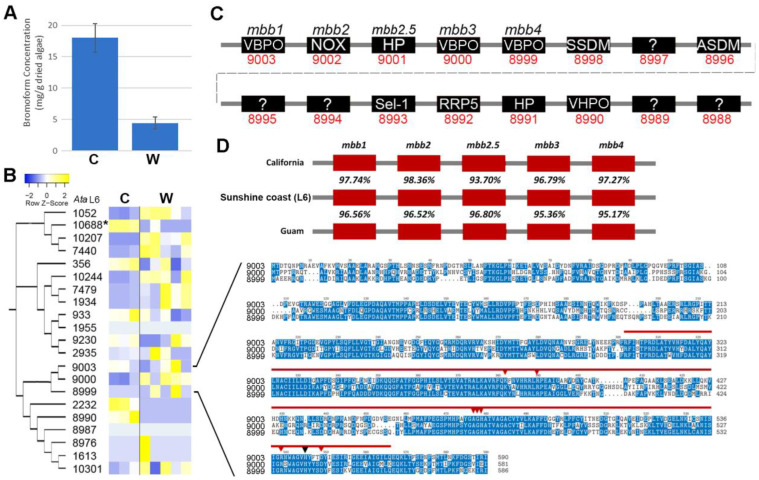
Comparative sequence analysis of *PAP2/VHPOs* in *A. taxiformis* (L6). (**A**) Bromoform concentration measured in the tetrasporophytes of cultured (C) and wild (W) *A. taxiformis*. Values correspond to mean % dry weight ± standard error (*n* = 3). (**B**) Phylogeny of *vBPO* genes and their relative level of gene expression (TPM log_2_) in cultured and wild *A. taxiformis* sporophytes. An asterisk (*) indicates significantly different (*p* < 0.05) and >4 FDR fold-change. Multiple sequence alignment shows VBPO protein sequences. The PAP2 domain is highlighted by the bold red line, and the red arrowheads point to active site residues. The black arrowhead denotes a histidine proposed to covalently bond to the vanadate cofactor [34]. (**C**) Schematic showing genomic organization of contig 5424 containing the *mbb* gene cluster in *A. taxiformis* (L6). This gene cluster includes a novel fifth gene within the *mbb* locus (*Ata*L6 9001) of uncharacterised function. VBPO, vanadium-dependent bromoperoxidase; NOX, NADPH oxidase; HP, hypothetical protein; SSDM, site-specific DNA-methyltransferase; ?, unknown function; ASDM, adenine-specific DNA methyltransferase; VHPO, vanadium haloperoxidase-like; Sel1, Sel1-repeat containing protein; RRP5, rRNA biogenesis protein 5. (**D**) Comparison of *A. taxiformis* (L6) *mbb* locus to those of the other two *A. taxiformis* lineages with available genome sequence data.

Halogenating peroxidases, or haloperoxidases (HPOs), are enzymes that oxidatively activate halides to the corresponding hypohalites at the expense of peroxides and are categorised into two groups, including (1) heme-dependent or (2) vanadium-dependent haloperoxides, which differ with respect to the prosthetic group and consequently in their catalytic mechanisms [37]. In seaweeds, numerous vanadium haloperoxidases (VHPOs) exist, while vanadium-dependent bromoperoxidases (VBPOs) have been of most interest due to their production of brominated compounds (e.g., bromine), which are assumed to be used in chemical-mediated defences in seaweeds [8]. Knowledge of how brominated compounds are biosynthesised could be used to improve their production [17]. We found that *A. taxiformis* (L6) tetrasporophytes maintained in culture produced higher levels of bromoform (18.02 ± 2.3 mg/g of dried algae) when compared to those in the wild (4.4 ± 0.96 mg/g of dried algae) (Figure 4A). A total of 21 genes identified in the *A. taxiformis* (L6) genome returned significant (e-value > 10^−3^) similarities with type 2 phosphatidic acid phosphatases (PAP2)/VHPOs. Phylogenetic analysis supported three predominant gene clusters, of which, 15 genes had at least some detectable level of expression in cultured or wild tetrasporophytes (Figure 4B). Only the VHPO gene, *Ata*L6 10688, showed a significant difference, being higher in cultured tetrasporophytes than in wild tetrasporophytes. The *Ata*L6 9003 gene was determined to have the highest overall expression of the 15 active VHPO genes identified via RNA-seq, but its level of expression was comparable between cultured and wild samples. The *Ata*L6 9003 gene corresponds to a VBPO previously described as *mbb1* of the *mbb* locus [17]. The *mbb* locus, known to consist of three VBPO and one NADPH-oxidase (NOX) gene, was identified in *A. taxiformis* (L6), with VBPO *Ata*L6 8990 identified to be proximal to *mbb1* (Figure 4C). A fifth gene within the *mbb* locus, denoted as *mbb2.5* (*Ata*L6 9001), was predicted and further confirmed to be present in the *A. taxiformis* California and Guam genomes (GenBank MN966723 and MN893468, respectively) (Figure 4D) [17]. Conservation of *mbb* locus genes between the sequenced *A. taxiformis* genomes indicated significant intra-specific variability for these genes of this locus (e.g., *mbb1* returned 96.56–97.74% sequence identity across the three genomes). It is possible that the variability of the *mbb* locus genes could result from neither the California nor Guam genomes being derived directly from the *A. taxiformis* Lineage 6 genome, although this cannot be ascertained from the data presented in [6]. Similarly, Lineage 6 of *A. taxiformis* has not been confirmed from California or Guam. In comparison, sequence analysis of the three VBPOs demonstrated that these proteins are moderately conserved and have similar lengths of between 581 and 590 amino acid residues. The *Ata*L6 *VBPOs* all featured a PAP2 domain, which is situated between the early-300 to the mid-500 amino acid residue positions and covered short regions of both high and low sequence conservation. In addition, all three *Ata*VBPOs contained eight highly conserved PAP2 active site residues, including a histidine (H) residue, to suggest that each *Ata*VBPO can form a covalent bond to the vanadate cofactor [38].

**Figure 5:**
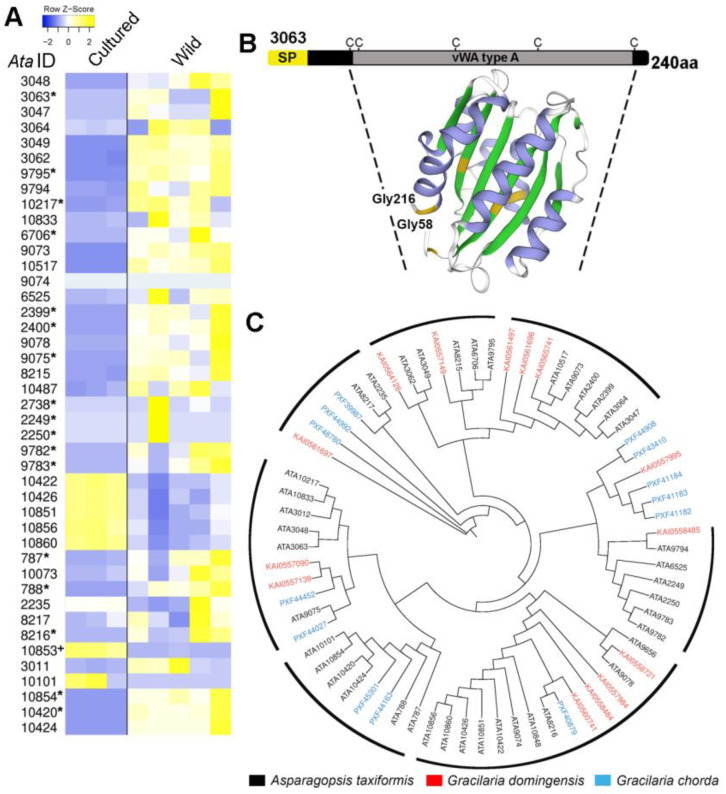
Identification and comparative analysis of *Asparagopsis taxiformis* (L6) rhodophyte VWA domain-containing proteins. (**A**) List of rhodophyte VWAs and their relative levels of gene expression (TPM log_2_) in cultured and wild tetrasporophytes. The asterisks (*) and the plus symbol (+) indicate significantly higher expression (*p* < 0.05) in wild and cultured samples, respectively, and with >4 FDR fold-change. (**B**) Schematic showing organisation of *Ata*L6 3063 and its basic protein structure. SP, signal peptide; vWA type A, vWA type A domain. (**C**) Phylogenetic tree of rhodophyte VWAs identified in *A. taxiformis* (L6), *G. chorda*, and *G. domingensis*. Seven clusters that include *A. taxiformis* (L6) are shown.

An *in silico* secretome analysis of the *A. taxiformis* (L6) genome-derived proteins, using conventional protein secretion pathway predictions [39], found that 332 proteins were likely secreted from cells (File S1f). This included an expanded family of proteins annotated as “collagen-alpha-like” proteins (~43; 12.95% of total proteins secreted), several of which were enriched (Figure 5) and demonstrated significantly higher levels of gene expression in wild sporophytes (Figure 5A). These proteins all shared the features of a signal peptide and at least one von Willebrand factor type A (VWA) domain with spatially conserved cysteine residues; a structure model indicated similar sequence identity (16.29%) to a proximal thread matrix protein-1 (PDB # 4cn9.2.A) found in the mussel byssus (Figure 5B and Figure S4) [40]. The proximal thread matrix protein 1 can bind collagen, thereby influencing the thread mechanical characteristics such as flexibility [41]. VWA domain-containing proteins are often associated with protein–protein interactions [42]. BLASTp similarity searches demonstrated some conservation (up to 62%) with predicted proteins from the red seaweeds *G. chorda* and *G. domingensis* (Figure 5C). Based on the aforementioned evidence, we suggest that these secreted VWA domain-containing proteins in *A. taxiformis* (L6), herein named rhodophyte VWAs, may help regulate the flexibility of the seaweed and/or mediate substrate attachment. It is important to note here that the cultured tetrasporophytes used in this study were not attached to a substrate. A secreted liquid mucilage is critical for seaweed bioadhesion, allowing for cell wall adhesion to a substrate, then for the cell wall to irreversibly harden post adhesion [43].

### 2.5. A. taxiformis Tetrasporophyte Proteomics

**Figure 6:**
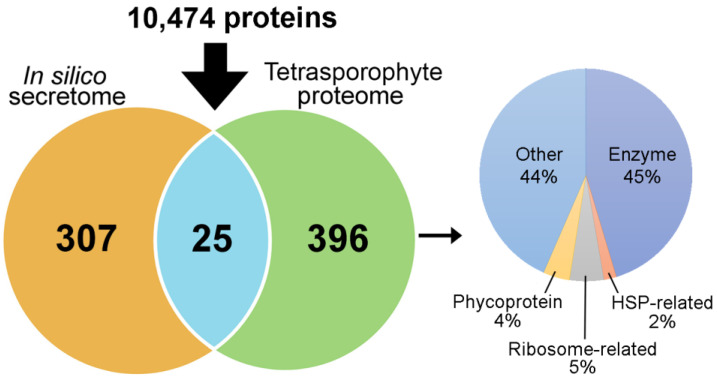
Venn showing *in silico* secretome of *A. taxiformis* (L6) genome-derived proteins (based on SignalP and TMHMM predictions) and tetrasporophyte identified proteins, also with distribution of functional categories.

The comparative gene expression analysis provided protein targets for further investigation, and their isolation (as native proteins) from seaweeds would enable such analysis, including structural and functional characterisation. For example, the *A. taxiformis* (L6) PAP2/VHPOs, animal heme peroxidase-like, and rhodophyte VWA proteins were of particular interest so their isolation would help for future functional characterisation to provide a more detailed understanding into their role in brominated compound biosynthesis and stress and/or defence responses. The *A. taxiformis* (L6) genome provided an excellent opportunity for proteomic investigation of this seaweed, with specific interest to isolate the secretome proteins stated above. Of the proteins predicted from the genome, 421 proteins were identified following tetrasporophyte extraction (via the use of Tris and Urea) and proteomic analysis (Figure 6 and File S3), of which 25 were predicted to be secreted, including a VHPO (encoded by *Ata*L6 1934), an animal heme peroxidase-like (*Ata*3164) and a rhodophyte VWA protein (AtaL6 10422). Besides putative secreted proteins, 45% of the extracted proteins consisted of enzymes (e.g., lipoxygenase, fructose-1,6-biphosphate aldolase, and peroxiredoxin), 4% phycoproteins (e.g., allophycocyanin beta 18 subunit, phycoerythrin gamma 31 kDa subunit, and phycobilisome linker polypeptides) and 44% that were either a hypothetical protein or a protein of unknown function.

This tetrasporophyte proteomic investigation of *A. taxiformis* (L6) will complement other experimental approaches to protein-based production in *A. taxiformis* (and other seaweeds), such as production of recombinant proteins (e.g., [17]) and gene/protein knockdowns. In addition, proteins that were annotated to enzymes were highly represented in the proteome; seaweed enzymes show prebiotic and nutraceutical potential, primarily through applications that develop bioactive compounds from seaweeds, as well as assist in the extraction and hydrolysis of macromolecules [44]. Also, phycobiliproteins (water-soluble pigments) are highly sought after as natural pigments of food, cosmetics, dyes, and other industries [45], so their purification and identification via a genomic resource database will assist the development of approaches for large-scale purification.

## 3. Materials and Methods

### 3.1. Asparagopsis taxiformis Tissue Collection

Triplicates of wild *A. taxiformis* tetrasporophytes were collected both on 24 February and 18 March 2021 from rocky reefs at Moffat Beach, Queensland, Australia (26°47′21.0″ S 153°08′37.5″ E), at a water depth of between 1 to 3 metres (m). Samples were collected with nitrile disposable gloves, rinsed with sterile seawater (SSW), placed in sterile centrifuge tubes, snap-frozen in a Taylor–Wharton CP300 Cryogenic Liquid Nitrogen (LN_2_) Shipper, and then stored in a laboratory freezer at −80 °C prior to use for DNA extraction. Additionally, a fresh portion of these samples were fixed in 4.0% paraformaldehyde in SSW for microscopic analysis.

Cultured *A. taxiformis* tetrasporophytes were grown vegetatively from tetrasporophytes collected at Moffat Beach (30 July 2019) and maintained under standard indoor cultivation conditions of a day/night light cycle of 16 hours (h) of light and 8 h of dark and a constant temperature of 22 °C. The tetrasporophytes were cultivated in aerated 2.0 litre (L) flasks with fortnightly SSW changes, along with the addition of F/8 enrichment medium. Healthy filaments were isolated at a small scale in 5 to 30 millilitre (mL) Petri dishes, and these were maintained with regular SSW exchanges (enriched with F/8) until April 2020. At this time, the cultured filaments were upscaled to 280 mL inverted culture vessels (“hatchers”) at a density of 1.0 grams per litre (g/L). Once upscaled to hatchers, cultures were maintained with weekly SSW changes (i.e., filtered and autoclaved SSW supplemented with 0.5 mL/L of F/4 medium and 448 micrograms per litre (µg/L) of germanium dioxide (GeO_2_) to prevent diatom growth. Cultured tetrasporophytes were then subsampled for microbial analysis on 24 February 2021 prior to processing for genomic analysis. Five subsamples were obtained from distinct culture vessels and snap-frozen in LN_2_ prior to microbial DNA extractions; then, fresh fragments were fixed in 4.0% paraformaldehyde in SSW for microscopic analysis.

### 3.2. Library Construction and Sequencing

*Asparagopsis taxiformis* cultured tetrasporophytes (2019 November collection, average temperature 23 °C) were selected as the most appropriate material for genome sequencing based on minimal microbial contamination (see section above). This sample of clonally propagated filaments was subsequently analysed using DNA barcoding with the *Cox2-3* spacer to confirm the cultured sample was *A. taxiformis* lineage 6 (L6), Great Barrier Reef strain [23], as were additional cultures collected and domesticated from the same site in 2018 and 2021. DNA extraction, amplification, and phylogenetic analysis followed procedures described previously [23,26].

For genomic DNA sample preparation, approximately (~) 20 g (g) fresh weight of a single-cultured sporophyte was harvested from hatcher vessels (28 January 2021) and snap-frozen in LN_2_. An approximately 5.0 g sample was shipped in dry ice to BGI, Hong Kong. Following nuclear DNA extraction via the standard protocols of BGI Genomics, a hybrid sequencing strategy was used for genome sequencing via combining both long reads from PacBio sequencing and short reads from BGI DNBSeq sequencing. Isolated genomic DNA was subjected to short-read library preparation with standard paired-end protocols. In brief, 1.0 micrograms (µg) of genomic DNA was randomly fragmented by Covaris, followed by fragment selection by Agencourt AMPure XP-Medium kit to an average fragment length of 300 to 400 base pairs (bp). Selected fragments were repaired and adenylated at their 3′ terminus, and then adaptors were ligated to the ends of each 3′ adenylated fragment. Each product was then amplified by PCR and purified using the Agencourt AMPure XP-Medium kit. The purified double-stranded PCR products were heat denatured into single strands, and these were then circularized by the splint oligo sequence. The single-strand circle DNA (ssCir DNA) were formatted as the final library and qualified by quality control. Subsequently, libraries with insert lengths of up to 370 bp were next constructed and sequenced using a MGISEQ-2000. As part of this sequencing process, each ssCir DNA molecule formed a “DNA nanoball (DNB)”, which contained more than 300 copies of each insert through rolling-cycle replication. The DNBs were loaded into the patterned nanoarray by using high-density DNA nanochip technology. Finally, 150 bp paired-end reads were obtained by combinatorial Probe-Anchor Synthesis.

To sequence longer reads, the high molecular weight DNA fraction of the *A. taxiformis* nuclear genome was sheared into fragments of approximately 20 kb using a Covaris g-tube (part No. 520079; KBiosciences, Maple Park, UK). The preparation and sequencing of the SMRTbell DNA library were performed according to the manufacturer’s protocols (Pacific Biosciences, Menlo Park, CA, USA). The SMRTbell library with 20 kb insert size was generated with the BluePippin system (Sage Scientific, Beverly, MA, USA). One lane was loaded and run on the Sequel System, which utilises single molecule, real-time (SMRT) sequencing with fluorescently labelled nucleotides.

### 3.3. Genome Assembly and Functional Evaluation

The DNBSeq generated a total of ~4.6 gigabases (Gb) of genomic data, which was composed of 31,146,070 raw reads. By filtering out low-quality reads, unknown nucleotides, adapters, and duplicated reads, a total of 29,530,652 clean reads with a GC content of ~44% were obtained. For pair-end reads, the k-mer frequencies (k = 17) were computed via the Jellyfish v2.2.10 dataset [46]. Finally, the *A. taxiformis* nuclear genome size was estimated based on the 17-mer depth distribution. In total, the PacBio sequencing approach generated ~11.8 Gb of data, which consisted of 1,137,468 polymerase reads. With >80x PacBio data, PacBio reads were firstly corrected and assembled using Canu (v1.7.1) [47]. Contigs were then polished using Pilon based on the short reads from DNBSeq [48]. The raw data from all sequencing approaches reported here was then submitted to the NCBI Bioproject and SRA database under a Bioproject accession number PRJNA809757. By using a sequence-based BLAST search approach, the published *A. taxiformis* mitochondria genome (NCBI accession number KJ398158.1) was compared to the assembly constructed here. The complete mitochondrial genome of *A. taxiformis* and the genes it encodes were then predicted. By running the same procedure on a complete *A. taxiformis* plastid genome (NCBI accession number NC_031148.1), we identified the putative plastid genes in our assembly.

Based on the assembly, overall statistics were calculated for GC content and median contig length. A Benchmarking Universal Single-Copy Orthologs (BUSCO 5.2.2) analysis was then performed to evaluate the accuracy and completeness of our assembly [49]. In general, BUSCO focused on core genes based on evolutionarily informed expectations of gene content of those conserved sequences. BUSCO relied on the Eukaryota_odb10 dataset from the OrthoDB database, which was used to construct single-copy gene sets of several large evolutionary branches. Via this approach, the BUSCO metric (% complete genes) can be used as a functionally complementary tool to the other technical metrics performed, such as median contig length N50.

### 3.4. Repeat Masking and Transposable Elements

Before we explored the functional fragments in the genome, specifically the protein-coding regions of the *A. taxiformis* nuclear genome, we examined the repetitive regions present in the assembly. By using RepeatModeler2 [50], a *de novo* repeat library was built. The repeat library, including known self-mobilizing transposable elements from other species, was then subjected to assessment by RepeatMasker (v 4.1.2-p1) [51] to find and mask homologous repeats in the assembled genome using RMBLAST (v 2.10.0+) as the default search engine. The total length of repetitive sequences was 100,684,772 bp, accounting for 70.67% of the assembled genome.

### 3.5. Gene Prediction and Functional Annotation

Since the BUSCO output is a set of highly conserved genes in our assembly that are single copy in most eukaryotic species, these sequences were used as a training dataset for the gene prediction tool, AUGUSTUS [52]. Briefly, AUGUSTUS (3.4.0) used the summarized features, such as the length distribution of single exons and the distribution of the number of exons per gene, to optimise gene prediction. By inputting the assembly after repeat masking, we are able to predict the protein-coding regions [52]. In total, the AUGUSTUS prediction identified 10,863 genes. Based on the predicted gene sequences and the translated protein sequences, we then ran the BLAST2GO pipeline to annotate and predict the putative function of each identified gene [53]. In brief, BLASTp was used to search the NCBI non-redundant protein databases [54] and Kyoto Encyclopedia of Genes and Genomes (KEGG) [55] with a threshold of E-value less than 1E^−5^. The top five BLASTp alignment results were used to extract the functional annotations. In addition to the BLASTp approach, the InterProScan tool was also utilised to identify all possible functional domains of each of the 10,863 proteins. To annotate the protein family information, HMMer was used to search all the Pfam protein family Supplementary Files [56].

### 3.6. Genome Decontamination

Since seaweeds, including *A. taxiformis*, coexist in symbiosis with their associated microbiome, any genomic data may contain microbial genomic contamination. In order to present a microbial contaminant-free genome, the predicted protein-coding genes were subjected to an *in silico* clean-up pipeline. Initially, the nucleotide sequences of the 10,863 protein-coding genes were cleaned using the FCS-GX tool [57] that searches for potential microbial contaminants using BLASTn against the NCBI non-redundant nucleotide database. However, it is important to note here that the majority of seaweed genomes currently deposited into this database have only been partially constructed to the scaffold level. When comparing gene sequences against considerably longer scaffolds, the resultant E-value may be low due to poor scaffold coverage. In our case, this resulted in several false positive results (i.e., a nucleotide sequence was falsely denoted as a contaminant). To prevent this, the translated open reading frames of those genes denoted as contaminants were subjected to further analysis using DIAMOND BLASTp [58] against a manually curated database, which contained all the reviewed Swiss–Prot amino acid sequences, as well as both reviewed and unreviewed (TrEMBL) amino acid sequences originating from algal groups rhodophyta, phaeophyceae, and chlorophyta [59] Following this analysis, only those genes that had a confident (E-value < 0.00001) top BLAST hit against a microbial subject (i.e., bacteria, archaea, and viruses) were classified as contaminants (*n* = 389) and removed (File S1g). Following this refinement, 10,474 genes were confidently predicted to be encoded by the *A. taxiformis* nuclear genome (File S1h).

### 3.7. Comparative Genome Analysis

To explore the common and unique functions of the *A. taxiformis* genes identified in our assembly, we first downloaded seven high-quality representative genomes and proteomes of red algae (*Cyanidiococcus yangmingshanensis, Cyanidioschyzon merolae, Galdieria sulphuraria, Porphyridium purpureum, Porphyra umbilicalis, Gracilariopsis chorda,* and *Chondrus crispus*) from NCBI based on the RefSeq category as a representative genome (File S1i). A comparative proteome analysis was then performed using OrthoFinder2. This provided a genome-wide comparison by grouping orthologous proteins. In brief, eight proteome sequences were used as input for OrthoFinder2 [60] to predict the orthologous relationship. The output from OrthoFinder2 includes the phylogenetic trees for thousands of detected orthologous groups. In OrthoFinder, these sequences were concatenated into a “supergene” and used for multiple sequence alignment by MAFFT [61]. Then, the consensus phylogenetic information from the orthologous groups was inferred by STAG algorithm [62]. The final unrooted species tree was built by FastTree using the maximum likelihood method [63]. Based on the species tree, the gene duplication events that were satisfied by the duplication-loss-coalescent model were extracted at each branch of the species tree. To obtain the basic functional information, we ran HMMer and extracted all the Pfam domain information for each of the seven proteomes [56]. For data analysis and visualisation, all statistical analyses were generated by R language. The basic bar plots were generated using the Excel chart function. The overlapping relationship of Pfam annotations and the heatmaps of the comparative genomics analyses were generated using in-house R scripts.

### 3.8. Targeted Gene Annotation

To predict transcription factors, we downloaded all known transcription factors from plant genomes in PlantTFDB version 4 [64] and then mapped our proteome to these. Finally, all the conserved transcription factor families were assigned based on the best match from BLAST output. Genome annotations were then used to target our investigation onto genes associated with (1) cell structure and regulation, (2) defence-related and stress genes, and (3) secreted proteins. The identification of genes in these groups was performed by using homologues identified in *C. crispus* [24] as BLASTp searches in the *A. taxiformis* (L6) genome (E-value cutoff 10^−3^). The *mbb* locus (involved in halogenated natural product synthesis) was identified through BLASTp for each *mbb* gene (GenBank MN966723). ExPASy Translate tool was used to identify the presence of open reading frames and introns across the *mbb* locus. The SignalP (Version 5.0) [65] and TMHMM (Version 2.0c) [66] tools were used to predict secreted peptides and proteins with a transmembrane domain. Multiple sequence alignments of the protein and nucleotide sequences were generated using Clustal Omega [67] in a ClustalW output format. NCBI Conserved Domains [68] were used to locate the domain and active site residues. Clustal Omega was utilised to determine nucleotide identity. Predicted structure homology modelling was performed using the SWISS-MODEL workspace [69].

### 3.9. Cultured and Wild Sporophyte Samples for Comparative Bromoform Analysis and RNA-Seq Gene Quantification

Cultured tetrasporophyte (filamentous, non-reproductive) of *A. taxiformis* (L6) were collected as described above. Wild tetrasporophytes (filamentous, non-reproductive) of *A. taxiformis* (L6) were also collected from Moffat Beach, Queensland, from subtidal depths of ~0.5 to 3.0 m (July 2020) for comparisons to the cultured strains. Wild sporophyte samples were transported in seawater to the laboratory of the University of the Sunshine Coast. Within 1 h, the samples were cleaned with sterile seawater to remove visible mud, epiphytes, and epibionts. All samples were then immediately snap-frozen in 1.5 mL microfuge tubes in LN_2_ and stored at −80 °C until use.

For gas chromatography-mass spectrometry (GC-MS) quantification of bromoform, bromoform was extracted from cultured and wild sporophytes (*n* = 3) by solid-liquid extraction of 0.02 g of dried, milled biomass into 4.0 mL of methanol, which contained a naphthalene internal standard, using the previously described method [70]. Samples were stored for 3 days at 4 °C prior to filtration and GC-MS analysis. Chromatography was performed on a Perkin Elmer Clarus 580 GC tandem Perkin Elmer Clarus SQ8S MS using a DB-5 column (Perkin Elmer Elite-5MS, 30.0 m × 0.25 mm × 0.25 µm). Injections (1.0 µL) were introduced to a 280 °C injection port with a 50:1 split ratio. The gas chromatography parameters were as follows: 40 °C for 1 min, increased to 250 °C at a rate of 20 °C/min, and immediately followed by a 30 second (s) equilibration time. The carrier gas used was helium, with a flow rate of 1.0 mL/min. The mass spectrometer analysed a mass range from 50 to 340 (*m*/*z*), from 3.0 to 12.0 min at 70 eV. Quantitation was conducted by referencing the TIC integrated by TurboMass software against analytical standard calibration curves. Final bromoform concentration did not account for the moisture content of biomass (typically ~3–5% under laboratory conditions).

For genome-guided RNA-seq quantification for cultured and wild tetrasporophytes, total RNA was extracted from the experimental samples (*n* = 8; 3 × cultured samples and 5 × wild samples), using a RNeasy Plant Mini Kit (QIAGEN, Hilden, Germany) according to the manufacturer’s protocol with the following modifications: 82 ± 8 mg of tissue was ground into a fine powder under LN_2_ before further lysis using Buffer RLT supplemented with 1.0% (*v*/*v*) β-mercaptoethanol. The extracted total RNA was treated with a DNA-free^TM^ DNA Removal Kit (Invitrogen, Carlsbad, CA, USA) to reduce genomic DNA contamination for cultured tetrasporophytes. RNA quality was measured using a NanoDrop2000 spectrophotometer (Thermo Scientific, Waltham, MA, USA) and the concentration and integrity verified using an Agilent RNA 6000 Nano assay run on an Agilent 2100 Bioanalyzer (Agilent, Lexington, MA, USA).

RNA samples (*n* = 8) were either sent on dry ice or stabilised for transport at ambient temperature by drying in GenTegra RNA tubes (GenTegra, Pleasanton, CA, USA) on a SpeedVac concentrator (Thermo Fisher Scientific, Waltham, MA, USA) according to the manufacturer’s protocol. Whole transcriptome sequence analysis by RNA-seq was performed by Novogene (Singapore) using Next-Generation Illumina sequencing, according to their standardised workflow. This process involved construction of cDNA libraries before sequencing of cDNA fragments using an Illumina NovaSeq 6000 System (raw sequence reads have been deposited in the NCBI GenBank PRJNA809757). Differential gene expression between cultured and wild tetrasporophyte RNA-seq data was mapped against the *A. taxiformis* (L6) genome using the CLC Genomics Workbench (Ver. 21.0.3) with default parameters. Transcript abundances were converted to transcripts per million (TPM) for each gene. Genes with significant upregulation or downregulation were identified using a significance cutoff set at a false discovery rate (FDR) *p*-value of <0.05. Differentially expressed genes (DEGs) underwent Gene Ontology (GO) annotation obtained using Blast2Go (E-value < 1.0E^−3^), InterProScan, and GO Mapping [71] performed on OmicsBox software, (Ver. 3.1). Venn diagrams were constructed to compare the total number of expressed genes between each replicate for the cultured and wild samples using the tool, Venn Diagram Maker (Good Calculators). A heatmap of DEGs from all of the cultured and wild replicates was constructed using ClustVis, utilising Z-Scores as input data [72].

### 3.10. Asparagopsis taxiformis Sporophyte Proteomics

Cultured tetrasporophyte samples (as described above) were snap-frozen and manually powdered with a mortar and pestle in LN_2_. For a single extraction, approximately 100 mg of the milled sample was homogenised in 20 mM Tris-HCl pH 7.4 buffer containing protease inhibitor (Merck, Bayswater, VIC, Australia) and then briefly vortexed and centrifuged at 4000× *g* for 25 min at 4 °C. The resulting supernatants and pelleted material were then extracted separately.

Initial supernatants were then further extracted by non-acetone and acetone procedures. For extractions without acetone, each supernatant was collected and processed through an Amicon Ultra-4 centrifugal filter unit (3 kDa cutoff; Millipore Corporation, Billerica, MA, USA) at 4000× *g* for 40 min (4 °C); then >3 kDa and <3 kDa samples were lyophilized in a vacuum concentrator (Express SpeedVac Concentrator SC250, Thermo Fisher Scientific). For extractions with acetone, the filtered supernatants were mixed with a pre-chilled (−20 °C) 100% acetone (volume = 4 times that of each protein sample) and stored overnight at −20 °C to allow for precipitation. Following centrifugation at 4000× *g* for 25 min at 4 °C, supernatants were decanted, and the protein pellets were air-dried to evaporate the acetone. Protein pellets were resuspended in lysis buffer (6.0 M urea), briefly vortexed, and then centrifuged at 4000× *g* for 25 min at 4 °C. As for the without acetone samples, the supernatant was collected and centrifuged through an Amicon Ultra-4 centrifugal filter unit (3 kDa cutoff); then >3 kDa and <3 kDa samples were lyophilised in a vacuum concentrator.

Lyophilised protein samples were resuspended in 0.1% (*v*/*v*) trifluoroacetic acid (TFA) in Milli-Q water and then processed by solid-phase extraction using a C_18_ Sep-Pak cartridges (500 mg sorbent per cartridge, 55 to 105 µm particle size; Waters), following the manufacturer’s instructions, to ensure complete removal of salts from all samples. Eluates were lyophilised in a vacuum concentrator (Express SpeedVac Concentrator SC250, Thermo Fisher Scientific) and stored at −20 °C prior to analysis. Samples extracted with acetone were trypsin-digested as previously described [73], following resuspension in 6.0 M urea. Samples were analysed by ultra-high-performance liquid chromatography (uHPLC) followed by mass spectrometry (MS) to identify proteins as per the previously described procedure [73]. A 6.0 µL aliquot of each sample was injected onto a 50 mm × 300 µm C18 trap column (Agilent Technologies, Mulgrave, VIC, Australia) and de-salted for 5 min using 0.1% (*v*/*v*) formic acid (aq) at 30 µL/min. The trap column was then placed in line with a 150 mm × 75 µm 300SBC18, 3.5 µm nano HPLC column (Agilent Technologies, Australia) for LC-MS analysis. Peptides were eluted at a flow rate of 300 nanolitres per min (nL/min) using a linear gradient of 1.0% to 40% solvent B (solvent A = 0.1% formic acid (*v*/*v*) in Milli-Q water; solvent B = acetonitrile: 0.1% formic acid (aq) 90:10) over 35 min followed by a steeper gradient from 40% to 80% solvent B over 5 min. The gradient was held at 80% solvent B for 5 min to wash the column and then returned to 1.0% solvent B for equilibration prior to injection of the next sample. The ion-spray voltage was set to 2400 V, declustering potential (DP) 100 V, curtain gas flow 25, nebuliser gas 1 (GS1) 12, and interface heater at 150 °C. The mass spectrometer acquired 500 ms full scan TOF-MS data, followed by 20 × 50 ms full-scan product ion data in an Information Dependent Acquisition (IDA) mode. Full-scan TOF-MS data was acquired over the mass range 350–1800 *m*/*z* and for product ion MS/MS 100–1800 *m*/*z*. Ions observed in the TOF-MS scan exceeding a threshold of 100 counts and a charge state of +2 to +5 were set to trigger the acquisition of product ion MS/MS spectra of the resultant 20 most intense ions. The data was acquired and processed using Analyst TF 1.5.1 software (ABSCIEX, Toronto, ON, Canada).

Proteins were analysed using PEAKS v7.0 (Bioinformatic Solutions Inc., Toronto, ON, Canada) against the protein database built from the reference *A. taxiformis* (L6) genome-derived protein database. *De novo* sequencing of proteins, database searches, and characterisation of specific post-translational modifications were used to analyse the raw data; FDR was set to ≤1.0%, and [−10 × log(*p*)] was calculated accordingly, where *p* is the probability that an observed match is a random event. The PEAKS used the following parameters: (i) precursor ion mass tolerance, 0.1 Da; (ii) fragment ion mass tolerance, 0.1 Da (the error tolerance); (iii) tryptic enzyme specificity with two missed cleavages allowed; (iv) monoisotopic precursor mass and fragment ion mass; (v) a fixed modification of cysteine carbamidomethylation; and (vi) variable modifications including lysine acetylation, deamidation on asparagine and glutamine, oxidation of methionine, and conversion of glutamic acid and glutamine to pyroglutamate. MS raw data was deposited into the PRIDE database under accession number PXD035669.

## 4. Conclusions

Our research has generated a high-quality reference genome for *A. taxiformis* (lineage 6) and identified substantial differences in gene expression patterns between tetrasporophytes collected from natural habitats versus those maintained in laboratory cultures. These differences were especially pronounced in genes associated with stress response mechanisms and defensive functions. Wild sporophytes exhibited upregulation of genes associated with heat shock proteins (HSPs), haloperoxidases, and oxidative stress responses, reflecting their adaptation to fluctuating environmental conditions. In contrast, cultured sporophytes showed enrichment in regulatory factors like kinases and TFs, likely due to their stable, controlled growth environment. Notably, the cyclin gene (AtaL6_9753), critical for cell cycle progression, was highly expressed in wild sporophytes but absent in cultured ones, suggesting suppressed reproductive development under laboratory conditions.

Our results also provided novel insights into the conservation and variability of the bromoform biosynthesis (*mbb*) locus across *A. taxiformis* lineages. The *mbb* locus, comprising *VBPO*s and a *NOX* gene, was identified in lineage 6, with *Ata*L6_9003 (*mbb1*) showing high expression in both wild and cultured tetrasporophytes. Comparative analysis revealed sequence variability in *mbb* genes among different *A. taxiformis* genomes (e.g., California and Guam lineages), suggesting lineage-specific adaptations in bromoform production. Thus, our study supports evidence of a biosynthetic pathway for bromoform in *Asparagopsis* that is driven by the VBPO proteins [74]. Besides VBPOs, our genomic analysis identified 21 genes with significant similarities to type 2 phosphatidic acid phosphatases (PAP2)/vanadium haloperoxidases (VHPOs), of which 15 showed detectable expression in tetrasporophytes. Our findings that cultured tetrasporophytes produced four times higher levels of bromoform than wild specimens further support the connection between these enzymes and bromoform biosynthesis, although additional research is needed to explicitly link these genes to the specific biochemical steps outlined in prior work. This enhanced understanding of the genetic and enzymatic mechanisms driving bromoform production in *Asparagopsis* could be leveraged to improve the production of these valuable brominated compounds.

Additionally, proteomic analysis uncovered novel secretory proteins, including rhodophyte von Willebrand factor A (VWA) domain-containing proteins, which may play a role in gland cell function and halogenated compound storage. These findings enhance our understanding of *A. taxiformis* biology and provide a foundation for optimising its cultivation for methane mitigation in livestock.

The genome sequencing of *A. taxiformis* (L6) has greatly enhanced the genetic resources available for this much-lauded seaweed, most well-known for its potential in mitigating methane production in ruminants and facilitating further comparisons between lineages. However, the potential applications of all lineages of *A. taxiformis* in agriculture and aquaculture are diversifying, with new research and development relating to enhancing fish production and boosting immunity [75], novel antimicrobial and biotechnology uses [76], as well as environmental applications with respect to understanding and utilising *A. taxiformis* where it has become a pest and invasive species [77]. More specifically, *A. taxiformis* is an invasive species within Europe and the Mediterranean Sea [78], whose impact on the resident ecosystem and coastal biodiversity is still debatable. For instance, the spread of *Asparagopsis* affects the composition, variability, and relative abundance of the associated macrofauna [79]. A recent microbiome and metabolomic-based study also indicated that the interaction between *A. taxiformis* and the coral *Astroides calycularis* could induce metabolic changes in both species. In another ecological example, *Asparagopsis* is associated with the distribution and abundance of peracarid crustaceans [80]. Therefore, future genomics-driven chemical ecology studies might provide insight to estimate system responses to macroalgal invasions based on the cascading effects of observed epifaunal assemblage changes.

The original commercialisation of bioactives from *Asparagopsis* related to extraction of antimicrobials for use in cosmetic and human care products was patented in the 1990s [81]. In addition, *A. taxiformis* has demonstrated potent antioxidant and cytotoxic activity, indicating its potential use of derived products as an anti-aging drug [82]. Moreover, it is a rich source of bioactive compounds such as iodine, lipids, carrageenans, and cellulose [75,83]. Finally, the *A. taxiformis* (L6) genome allows for more robust comparative evolutionary comparisons and assessment of genome innovations in seaweed.

To facilitate precision genetic manipulation of this seaweed for large-scale aquaculture production, we have shared our high-quality draft genome assembly, which includes well-annotated gene and protein sequences, which can be used for future in-depth research of the red seaweeds. This could include the use of modern molecular techniques such as CRISPR-catalysed mutagenesis for the targeted manipulation of specific biosynthesis pathways (e.g., the bromoform biosynthesis pathway) in *A. taxiformis*. Furthermore, due to the significant variability between *A. taxiformis* lineages, there also exists the potential for the application of advanced genomic tools such as Nanopore technology to further characterise both the DNA and RNA landscapes of individual lineages. Similarly, our transcriptomic comparison of gene expression, including the expression of TF encoding genes, in cultured and wild *A. taxiformis* (L6) tetrasporophytes revealed considerable differences in the level of expression of individual genes in these two samples originating from dramatically different growth environments. In plants, including the genetic model plant species *Arabidopsis thaliana* (*Arabidopsis*), small non-coding RNAs (sRNAs), including the microRNA (miRNA) class of sRNA, have been demonstrated to act as master regulators of TF gene expression as part of the adaptive or defensive response of a plant to environment stress or invading pathogens, respectively [84,85,86]. Thus, the availability of a genome sequence will enable the future identification of *A. taxiformis*-specific miRNAs involved in stress responses and/or pathogen defence, and these novel miRNA species could potentially be used as the basis of the development of artificial miRNA (amiRNA) technology for use in *A. taxiformis*, and other red seaweed species, for highly specific gene expression manipulation [87,88]. At present, across the globe, significant investment has led to the farm-level demonstration of the utility of *A. taxiformis* as an aquacultural species. Therefore, the timely delivery of this database resource could further identify *A. taxiformis* (L6) as an aquacultural species of importance, and via the functional characterisation of genes associated with bromoform synthesis, stress responses, pathogen defence, and reproduction, new and exciting avenues for marker-assisted seaweed breeding will be identified and used in the future to generate novel *A. taxiformis* lineages that meet the demands of the consumer market.

## Figures and Tables

**Table 1:** Overall statistics for *A. taxiformis* (L6) genome assembly.

Assembly Statistics	
Total base pairs (bp)	142,472,235
Number of contigs	3308
Average contig length (bp)	43,069
Largest contig length (bp)	523,412
N50 (bp)	55,006
Gaps	0
**BUSCO overview**	
Overall coverage (C/Total)	81.60%
Complete BUSCOs (C)	208
Complete and single-copy BUSCOs (S)	183
Complete and duplicated BUSCOs (D)	25
Fragmented BUSCOs (F)	11
Missing BUSCOs (M)	36
Total BUSCO groups searched	255

**Table 2:** Summary of *A. taxiformis* (L6) genes involved in defence-related and stress-related processes. Number of corresponding genes significantly upregulated (>4-fold) in cultured or wild is shown. For more details, see File S2.

Category	Number	Examples	Cultured	Wild
Halogen metabolism	49	PAP2/vanadium haloperoxidases, non-animal heme peroxidases	1	5
Potential pathogen receptors	391	WD40-repeat containing proteins, LRR and TRR domain proteins, Sel1 repeat-containing proteins	8	6
Potential defence effectors	8	SGT1 ortholog, apoptosis-inducing factor, exportin	0	0
Stress genes ROS scavenging	27	Ascorbate peroxidase, Cu/Zn superoxide dismutase, glutathione reductase, peroxiredoxin	4	1
Stress genes heat shock proteins	59	HSP20, HSP40, HSP90, HSP100, HSP70 binding protein, heat shock transcription factor	4	7
Stress-related genes	59	T-complex alpha/beta/gamma, tubulin binding complex B/C/D, peptidyl-prolyl cis-trans isomerase	4	5
Cytochrome P450 (CYP450)	13	CYP51G, CYP97G, CYP80B	3	0

## Data Availability

Raw data of all genome sequencing was submitted to NCBI BioProject and SRA database under a BioProject accession number PRJNA809757. Phylogenetic identification data was submitted under GenBank accession number OP779373. Mass spectrometry peptide raw data was deposited into the PRIDE database under accession number PXD035669. The genome assembly can also be accessed via University of the Sunshine Coast at https://doi.org/10.25907/00708.

## Supplementary Materials

The following supporting information can be downloaded at: https://www.mdpi.com/article/10.3390/plants14101523/s1, File S1: (a) Overall statistics for *Asparagopsis* genomes. (b) Genome annotation, including gene ID, protein description, length, and sequence. (c) Summary of mitochondrial and plastid genes predicted from their genomes. (d) Transcription factors in *Asparagopsis taxiformis* (L6). (e) Differential gene expression between cultured and wild sporophytes. (f) In silico secretome. (g) List of the taxonomy IDs for all Asparagopsis taxiformis (L6) genomic sequences classified through the miniKraken database. Yellow, genes which were excluded from final genome. (h) Genome annotation, including gene ID, protein description, length and sequence. (i) Summary of red algae genomes used for comparative analysis; File S2. List of *Asparagopsis taxiformis* (L6) genes involved in defence and stress; File S3. Sporophyte proteome; Figure S1: Phylogenetic analysis of *A. taxiformis* used in this study, based on cox2-cox3 spacer; Figure S2: Summary of (A) *A. taxiformis* (L6) gene BLAST match identity, (B) number of genes for the top ten most abundant gene ontology terms in cellular component, biological process, and molecular function, and (C) summary of *A. taxiformis* (L6) gene InterPro annotations; Figure S3: Principal component scatter plot for RNA-seq of culture (C) and wild (W) sporophyte; Figure S4: SWISS-MODEL structure predication for protein encoded by *A. taxiformis* (L6) gene 3063, using a proximal thread matrix protein (PDB # 4cn9.2.A) as template.

## Abbreviations

The following abbreviations are used in this manuscript:

GO	Gene ontology
HSP	Heat shock protein
L6	Lineage 6
NOX	NADPH-oxidase
NR	Non-redundant
PAP2	Type 2 phosphatidic acid phosphatases
rRNA	Ribosomal RNA
SSW	Sterile seawater
TF	Transcription factor
TTF	Trihelix transcription factor
VBPO	Vanadium-dependent bromoperoxidase
VHPO	Vanadium haloperoxidases
VWA	von Willebrand factor type A
