# Supplementary material for: Insights into the Red Seaweed Asparagopsis taxiformis Using an Integrative Multi-Omics Analysis
Source: Plants (Basel). 2025 May 19;14(10):1523. doi: 10.3390/plants14101523 (PMC12115072; doi:10.3390/plants14101523)
Supplement: Supplementary file 1 [file plants-14-01523-s001.zip › Supplementary Figures.pdf]

Supplementary Figures

Figure S1. Phylogenetic analysis of *A. taxiformis* used in this study, based on *cox2-cox3* spacer.

Figure S2. Summary of (**A**) *A. taxiformis* (L6) gene BLAST match identity, (**B**) Number of genes for the top ten most abundant gene ontology terms in cellular component, biological process and molecular function, and (**C**) Summary of *A. taxiformis* (L6) gene InterPro annotations.

Figure S3. Principal component scatter plot for RNA-seq of culture (C) and wild (W) sporophyte.

Figure S4. SWISS-MODEL structure predication for protein encoded by *A. taxiformis* (SC) gene 3063, using a proximal thread matrix protein (PDB # 4cn9.2.A) as template.

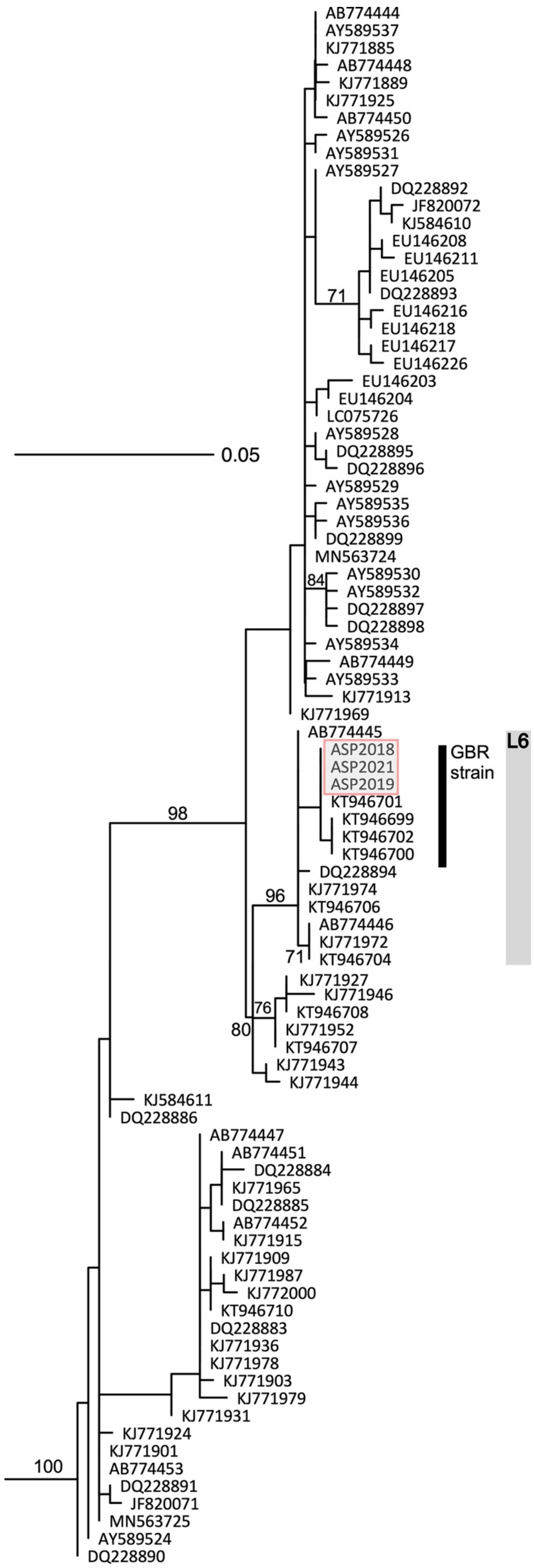

Figure S1. Phylogenetic analysis of *A. taxiformis* used in this study, based on *cox2-cox3* spacer.

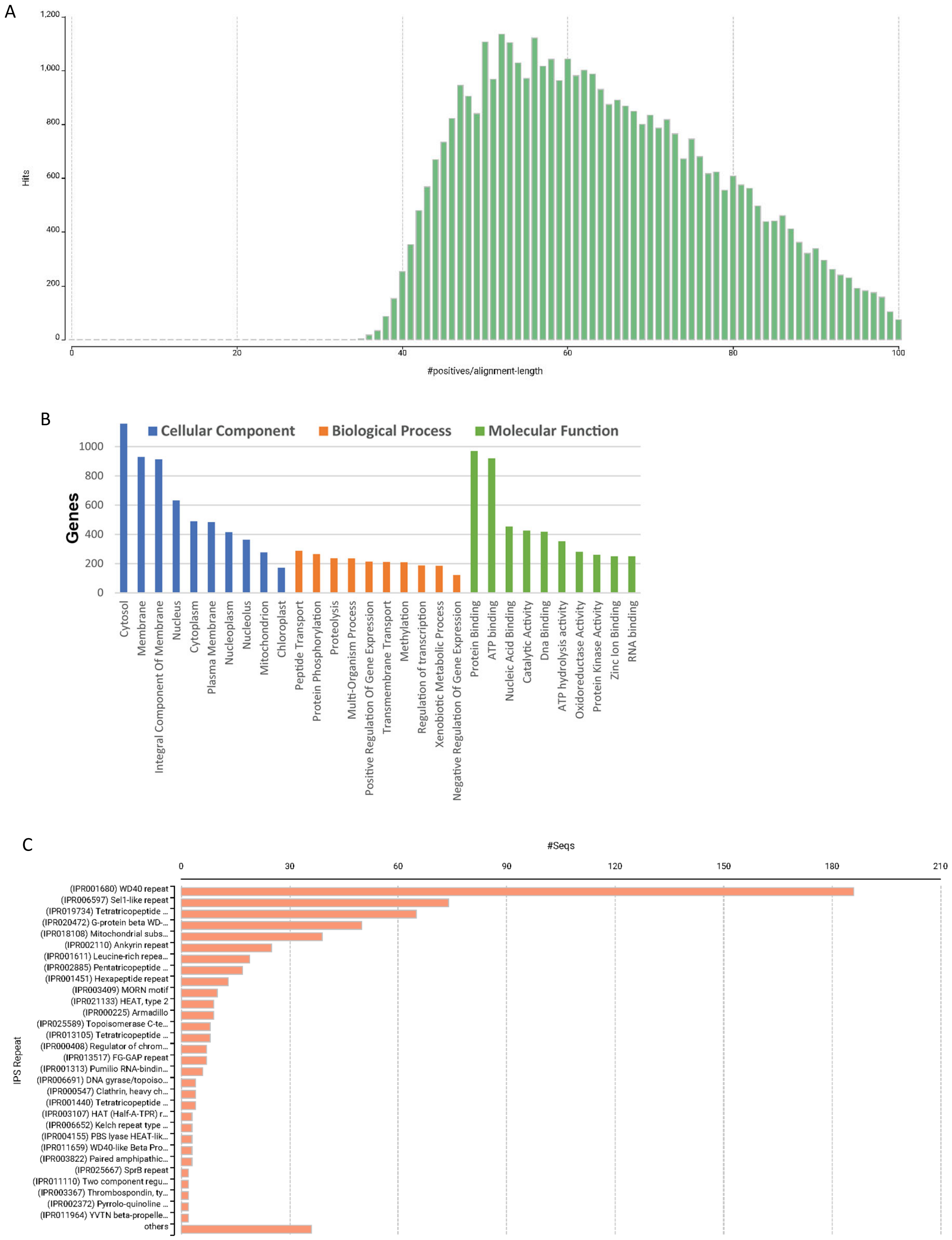

Figure S2. Summary of (**A**) *A. taxiformis* (L6) gene BLAST match identity, (**B**) Number of genes for the top ten most abundant gene ontology terms in cellular component, biological process and molecular function, and (**C**) Summary of *A. taxiformis* (L6) gene InterPro annotations.

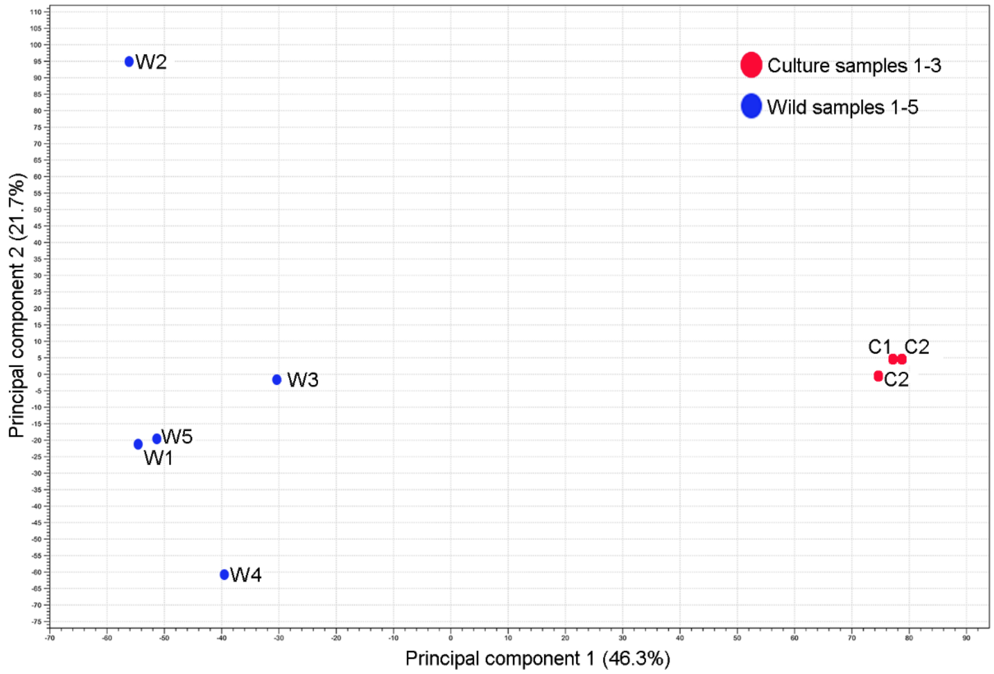

Figure S3. Principal component scatter plot for RNA-seq of culture (C) and wild (W) sporophyte.

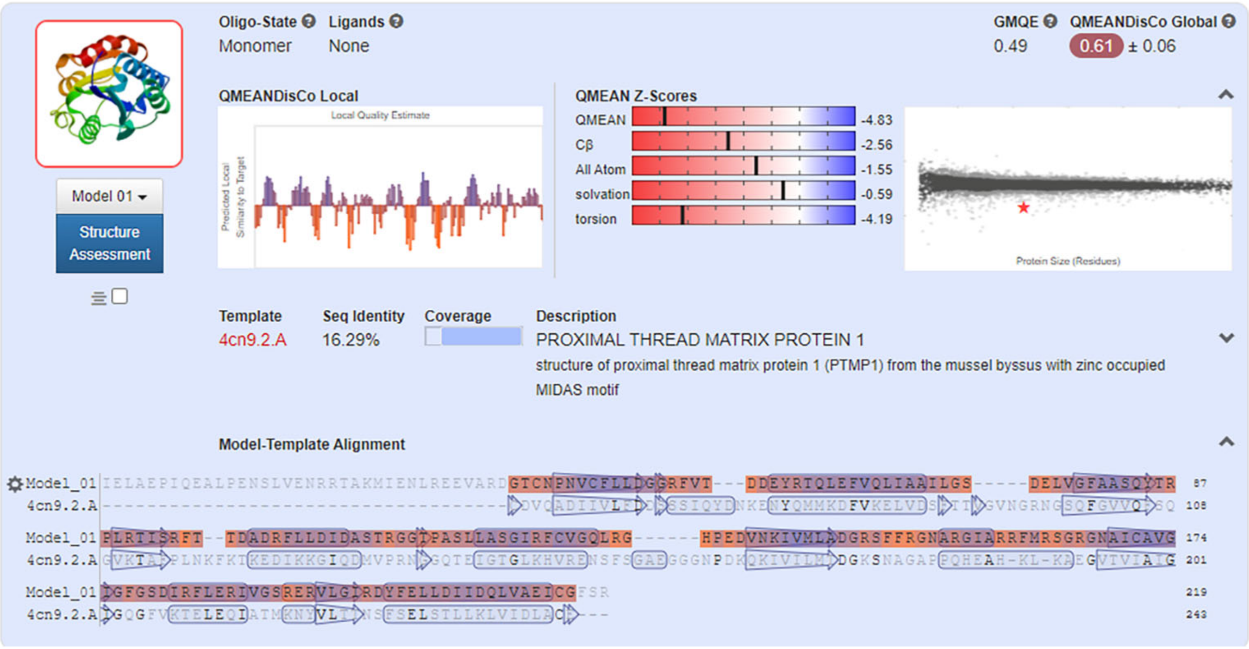

Figure S4. SWISS-MODEL structure predication for protein encoded by *A. taxiformis* (SC) gene 3063, using a proximal thread matrix protein (PDB # 4cn9.2.A) as template.
